# Tree failure – A natural phenomenon with forensic implications

**DOI:** 10.1007/s12024-024-00858-9

**Published:** 2024-07-19

**Authors:** Roger W. Byard

**Affiliations:** 1 Pathology, Forensic Science SA, Adelaide, South Australia Australia; 2 School of Biomedicine, The University of Adelaide, Level 2, Room N237, Adelaide, South Australia 5005 Australia

**Keywords:** Tree-related deaths, Tree failure, Summer branch drop, Fatalities, Blunt force trauma

## Abstract

Tree failure, or summer branch drop, refers to situations where large branches or whole trees may unexpectedly drop on unsuspecting victims. On occasion, this involves vehicles impacted by falling overhanging trees or branches. To study this phenomenon further review of the Forensic Science SA, Adelaide, Australia, database and an internet news search were undertaken over a 20 year period from March 2004 to February 2024 for all cases where unexpected accidental deaths in South Australia had been caused by falling branches or trees. There were six cases (age range 12–63 years; average 39 years; M:F 1:2). Three deaths occurred when branches dropped onto vehicles being driven along roads, 2 occurred when entire trees dropped onto the victims (one in windy weather) and 1 was due to a branch drop. Three tree species were identified as a grey box gum tree (*Eucalyptus microcarpa*), a lemon-scented gum tree (*Corymbia citriodora*) and a Cottonwood tree (*Populus deltoides*). The remainder were variants of eucalyptus (gum) trees. Deaths due to tree failures are uncommon and may involve a variety of different tree species with these events not necessarily occurring during high winds or storms. The major findings at autopsy are blunt force injuries.

## Introduction

Trees are integrally associated with human life and activities and are present in most parts of the inhabited planet in urban, rural and wilderness areas. Fatalities involving trees may arise from a wide variety of scenarios (Table 1) that involve work, recreational, psychological and accidental circumstances. The following analysis provides an overview of tree-related deaths with a specific focus on the subset of unexpected accidental deaths due to so-called ‘tree failures’ where lethal injuries have resulted from either large branches detaching from mature trees or from trees uprooting. Tree failures involve root failure, stem failure, branch failure or various combinations of these [1].

**Table 1:** Situations where fatalities have been associated with trees

Impact from falling branches/trees/cones-nuts
Tree felling/sawing
Arborist activities
Falling out of trees
i. Work related
ii. Recreational
Vehicle impacts with trees
i. Accident
ii. Suicide
Hanging from trees
i. Suicide
ii. Homicide
Bush/brush fires
Hunting using tree stands
Animal/insect-related
Toxins/poisons

## Materials and methods

Review of the Forensic Science SA, Adelaide, Australia, database and an internet news search were undertaken over a 20 year period from March 2004 to February 2024 for all cases where unexpected accidental deaths in South Australia had been caused by falling branches or trees. The age, sex and circumstances of the accidents, including the species of tree, were recorded. Identifying features such as specific injuries were not documented. Cases where deaths were due to tree felling or sawing, falling from trees, vehicle impacts into trees, hanging from trees, bush/brush fires, animal/insect activity or to toxins or poisons, were excluded from the study.

## Results

**Fig. 1:**
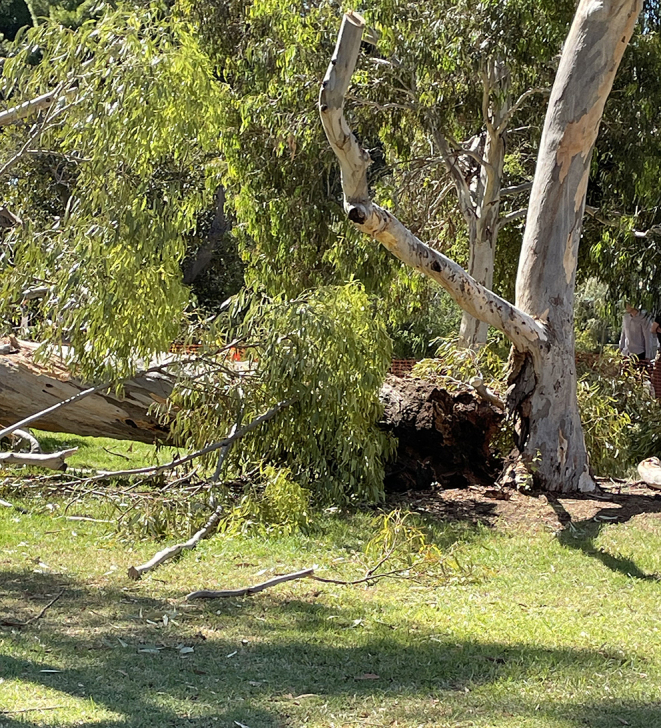
A large gum tree in public parklands in Adelaide, South Australia, which had split at the base and fallen crushing a 22-year-old woman (case 3). The image shows rotting of the base

There were six cases which consisted of:

1. A 12-year-old girl who was playing in a community church yard in high winds who was struck by a falling 10 m high eucalyptus (gum) tree [2].
2. A 20-year-old woman who was struck while driving her car by a 7 m long branch overhanging a road that fell from a grey box gum tree (*Eucalyptus microcarpa*) [3].
3. A 22-year-old woman who was sitting under a tree in shade who was struck by a falling section of eucalyptus tree that had split from the main trunk. It was estimated to weigh between 8 and 10 tonnes (Fig. 1). There was no significant wind at the time [4].
4. A 57-year-old man who was struck by a falling branch from a lemon-scented gum tree (*Corymbia citriodora*) in his back yard [5].
5. A 59-year-old woman who was struck while driving her car by a large branch overhanging a road that fell from a Cottonwood tree (*Populus deltoides*) [6].
6. A 63-year old man who was struck while driving his car by a branch overhanging a road that fell from a eucalyptus tree [7].

All victims died of blunt force trauma injuries.

## Discussion

Work-related deaths associated with trees occur most often with tree felling and lopping, logging, sawmilling and arborist activities [8]. Logging and forestry work is inherently dangerous with significant rates of serious injury and death. More than 100 deaths per 100,000 person years are reported, which is 2–3 times greater than other high-risk occupations such as mining and construction [9–11]. Tree lopping may cause injury and deaths amongst arborists and also amongst amateur gardeners, who tend to be in an older age bracket (40 vs. 60 years on average). Electrocution and chainsaw injuries are also possible [12].

Falls from trees may be either work-related or recreational. Work fatalities from falls include not only descent from trees but also from ladders and boom lift buckets [12]. Trees may be involved in lethal vehicle crashes and it may be difficult in certain cases to determine whether the impact was inadvertent or intentional. In the absence of documented suicidal ideation and/or a note, careful assessment of the crash scene looking specifically at weather and road conditions and for the presence or absence of skid marks or attempts to avoid the impact may provide important information to evaluate possible intent [13].

Trees may provide convenient attachment points for ligatures in suicidal hangings and have the convenience sometimes of being located in secluded places to facilitate the activity [14]. In such cases it must be remembered that hangings may also be used to disguise homicides [15, 16] as occurred in one of the victims of the infamous Snowtown killings in South Australia who was found hanging from a tree at Humbug scrub [17].

Deaths involving trees may also occur with bush/brush fires, during hunting using tree stands, and also from animal or insects resident in trees or from tree derived toxins or poisons [8, 18, 19]. Impact may occur from falling seeds such as coconuts which may hit the head with considerable force [20].

Finally deaths may be associated with so-called tree failures, or summer branch drop, where large branches or whole trees may unexpectedly descend [21]. As can be seen from the reported cases, lethal episodes may occur when individuals are merely standing or sitting underneath tress, or are driving along roads with overhanging branches. The size and weights of the branches or trunks are such that the episode often occurs extremely quickly usually precluding escape.

Windy conditions and storms, with or without ice or snow accumulation, may predispose to tree falls (reported in 90% of fatalities [22]). This includes thunderstorms, cyclones and tornadoes [23, 24]. This is not always the situation as fire, drought and insect activity also predispose to failure. It is sometimes the case, however, that an apparent spontaneous dropping of a branch may actually represent a branch that was broken off during a previous storm that was trapped in the canopy, only to subsequently fall. These have been called ‘widow makers’ [8]. Predicting the likelihood of tree failure before and during storms has proven challenging [1, 25, 26].

**Fig. 2:**
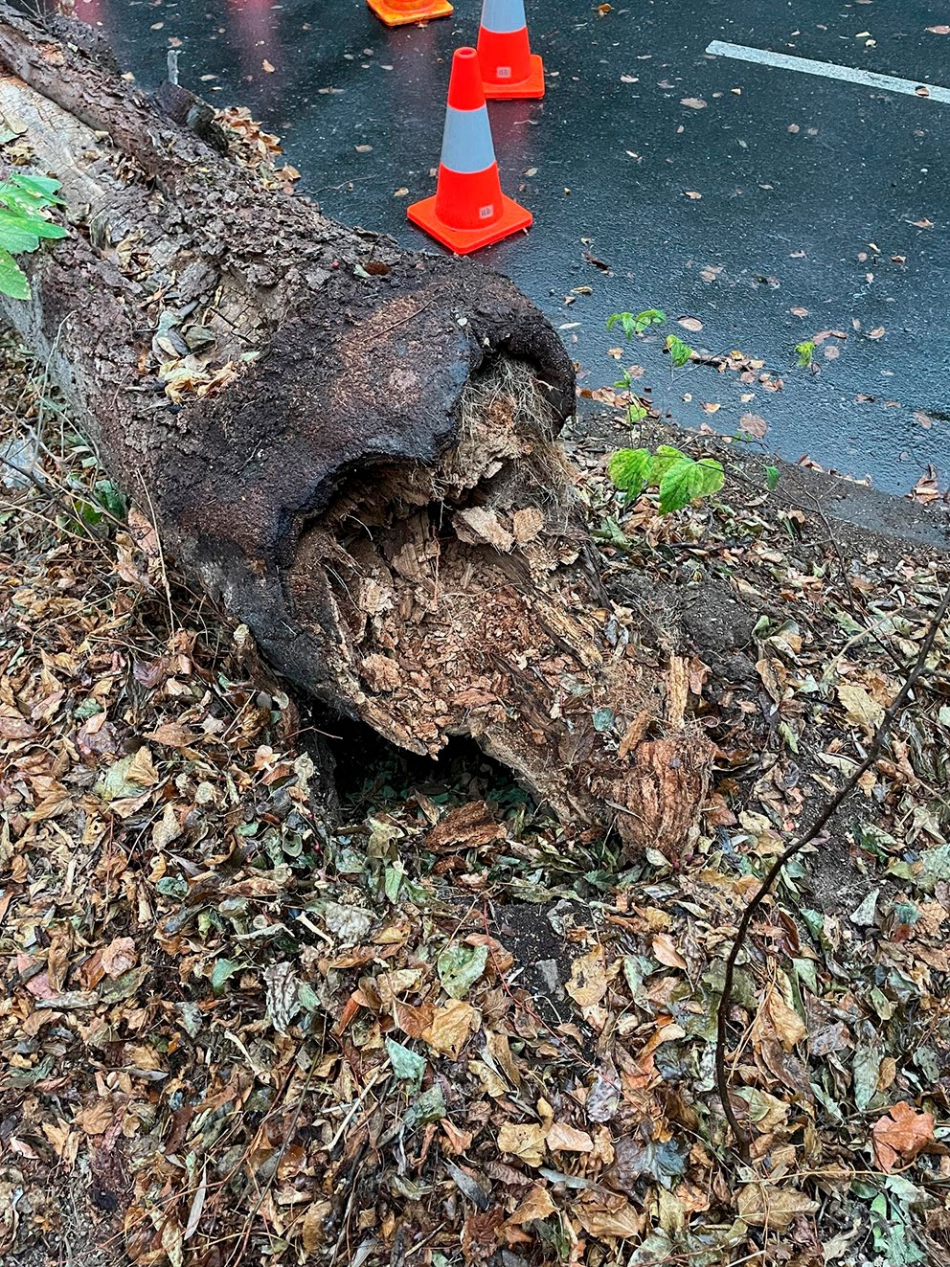
A street tree in nearby North Adelaide which suddenly collapsed shortly after the incident described in case 3 narrowly missing parked cars. The tree is a hackberry (*Celtis occidentalis*) and has also rotted through at the base

A wide variety of trees may suffer from failure, the causes of which include auto-amputation where trees preferentially drop branches to conserve moisture in periods of hot weather, and collapse from rotting at the base (Figs. 1 and 2). Branch drop may occur following heavy rain with internal degradation of trees often being difficult to detect. In urban situations soil compaction and reduced light may accelerate tree decline [21]. In one study half of deaths reported during outdoor recreational activities occurred when a branch or tree fell onto a tent [27].

The findings at autopsy depend on the circumstances, and range from severe multiple injuries involving all body cavities to blunt head trauma. In some cases there may be evidence of crush asphyxia [12, 28, 29].

As can be seen from the data on lethal cases accrued over a 20-year period in the current study these events are not common. For example, Schmidlin reported only 407 deaths from wind-related tree failures in the United States from 1997 to 2007 [23] with 273 deaths over 160 years in Australia [22]. In the United Kingdom 64 deaths occurred between 1999 and 2009 from falling trees or branches, representing an average of 6.4 deaths per year [30]. In a clinical setting it was estimated in one report that only 0.26% of hospital admissions for trauma over an 8.5-year period involved trees and that only 0.15% were associated with tree failure [31].

This study has demonstrated that deaths due to tree failures, although uncommon, may be regularly seen in forensic practice. A variety of different tree species may be involved and these events may not necessarily occur during high winds or storms. The major findings at autopsy are blunt force injuries, sometimes with evidence of crush asphyxia.

## Key Points

1. Tree failure, or summer branch drop, refers to situations where large branches or whole trees unexpectedly drop onto victims.
2. A 20 year review of cases in South Australia identified 6 deaths (age range 12-63 years; average 39 years; M:F 1:2).
3. Deaths occurred when branches dropped onto vehicles, or when entire trees or branches dropped.
4. Deaths due to tree failures are uncommon, may involve a variety of different tree species and occur not necessarily during high winds or storms.
